# CRUSADE Score is Superior to Platelet Function Testing for Prediction of Bleeding in Patients Following Coronary Interventions^☆^

**DOI:** 10.1016/j.ebiom.2017.05.010

**Published:** 2017-06-01

**Authors:** Junghee Bang, Sun Young Choi, Moo Hyun Kim, Victor Serebruany

**Affiliations:** a Department of Cardiovascular Surgery, Dong-A University Hospital, Busan, South Korea; b Heart and Brain Clinical Center, Dong-A University Hospital, Busan, South Korea; c Department of Cardiology, Dong-A University Hospital, Busan, South Korea; d HeartDrug™ Research Laboratories, Johns Hopkins University, Baltimore, MD, USA

**Keywords:** Bleeding, VerifyNow Analyzer, CRUSADE score, Dual antiplatelet therapy, Prediction

## Abstract

Hypothetically, diminished platelet reactivity (PR) during dual antiplatelet therapy (DAPT) should cause extra major bleeding events (MBE), although definite evidence is lacking. Multiple scores have been proposed to stratify bleeding risk, but their predictive value during DAPT is unclear. We compared the performance of the Can Rapid Risk Stratification of Unstable Angina Patients Suppress Adverse Outcomes with Early Implementation of the ACC/AHA Guidelines (CRUSADE) with PR testing to predict MBE in Korean patients with acute coronary syndrome. We screened 1105, and included 903 consecutive patients who underwent coronary interventions. All patients received DAPT, while MBE were assessed by BARC scale. Admission platelet reactivity was assessed with VerifyNow Analyzer simultaneously with CRUSADE score, and MBE were collected at 1 month and at 1 year post stenting. There were a total of 113 (11%) MBE at 1 month, and extra 41(5%) MBE at 1 year. At 1 month MBE prediction was superior by CRUSADE score (AUC: 0.816, 95% CI: 0.79 0.84, p < 0.0001), compared to PR (AUC: 0.605, 95% CI: 0.572–0.637, p = 0.0007). Moreover, CRUSADE score remains the independent predictor of MBE by multivariate analyses (OR = 2.94, 95% CI: 2.18–3.96, p < 0.0001). At 1 year MBE also correlated, but were not significantly different between admission CRUSADE score (AUC: 0.62, 95% CI: 0.58 0.66, p = 0.0183) and PR (AUC: 0.674, 95% CI: 0.63–0.71, p = 0.002). We conclude that MBE are more common in real life than reported in clinical trials. CRUSADE score was superior to PR testing for predicting short-term, but not 1 year MBE in Korean patients undergoing percutaneous coronary intervention and treated with DAPT.

## Highlights

• Dual antiplatelet therapy for 1 year is a golden standard following coronary stenting in patients with acute coronary syndromes.
• Bleeding is a life-threatening major complication associated with chronic aggressive antiplatelet strategies.
• Predicting bleeding based on clinical features or established biomarkers is upmost yet unsolved mystery affecting clinical outcomes in such high-risk patients
• The CRUSADE clinical score predicts bleeding better than platelet reactivity indices
• Short-term bleedings at 30 days, but not delayed 1-yearly hemorrhages were better predicted by CRUSAIDE in East Asians.

Dual antiplatelet therapy for 1 year is common following coronary stenting in patients with acute coronary syndromes. However, bleeding is a life-threatening major complication and shortcoming associated with chronic aggressive antiplatelet strategies. CRUSADE score was introduced for better prediction of in-hospital bleeding, while delayed bleeding risks remained unclear. Usually bleeding risk is assessed based on clinical features or by established biomarkers We tested the hypothesis on the superiority of CRUSADE score versus conventional platelet reactivity in a large Korean registry, and revealed the following: major bleeding events in real life are more frequent, and are heavily unreported in clinical trials; the CRUSADE clinical score predicts bleeding better than platelet reactivity indices. Unfortunately, short-term bleedings at 30 days, but not delayed 1-yearly hemorrhages were better predicted by CRUSADE in East Asians.

## 1 Introduction

Predicting major bleeding events during dual antiplatelet therapy (DAPT) with aspirin and P_2_Y12-inhibitor combination is currently one of the top priorities and unsolved mysteries in modern cardiology (Windecker et al., 2014). Unfortunately, there is no “sweet spot”, or “comfort zone” for optimal platelet inhibition challenging uniformed DAPT regimens. In fact, individual patients vary greatly with regard to threshold for bleeding events, and impact of residual platelet reactivity while on DAPT is not linear, or necessarily predictive of catastrophic hemorrhages. Importantly, DAPT downgrade dose/regimen adjustments in some patients with higher bleeding risks is currently not recommended by guidelines (Steg et al., 2011) or the FDA (NDA 294-886, 2013, Medical reviews on Vorapaxar). Another critical issue is the discrepancy between low bleeding rates picked up in clinical trials with the “real life” clinical experience. Indeed, major randomized trials report very few bleeding events by deliberately applying conservative exclusive bleeding scales artificially diminishing the caliber of the problem (Wiviott et al., 2007, Wallentin et al., 2009, Tricoci et al., 2012). This discrepancy is especially alarming since bleeding has been recently recognized as a challenge for survival (Généreux et al., 2015, Aradi et al., 2015).

The CRUSADE (Can Rapid Risk Stratification of Unstable Angina Patients Suppress Adverse Outcomes with Early Implementation of the ACC/AHA Guidelines) bleeding score has been recently introduced to predict bleeding in non-STEMI patients (Subherwal et al., 2009). A patient's CRUSADE Bleeding Score equals the sum of the weighted scores for the independent predictors (female sex, history of diabetes, peripheral vascular disease), admission clinical variables (heart rate, systolic blood pressure, signs of CHF), and admission laboratory values (hematocrit, calculated creatinine clearance), and ranged (1–100 points). Originally, CRUSADE considers likelihood of having an in-hospital early major bleeding event. Later research validated CRUSADE durability to 30 days, and even 1-year hemorrhagic risks, and expanded non-STEMI cohort to all post-PCI patients on DAPT (e.g. Al-Daydamony and Farag, 2016, Li et al., 2016).

Indeed, low residual platelet reactivity while on DAPT may be linked to greater bleeding risks (Brar et al., 2011), however, the quality large uniformed datasets matched with CRUSADE are still lacking. We assessed simultaneous admission CRUSADE score with platelet reactivity for predicting major bleeding in a large cohort of post-stenting patients of Korean descent.

## 2 Methods

### 2.1 Patients

Between November 2008 and November 2015, the total of 1105 patients were prescreened, and 903 post-PCI patients qualified (Dong-A University Medical Center, Busan, Korea) receiving maintenance DAPT (75 mg/day clopidogrel, or 10 mg/day prasugrel, or 180 mg/day ticagrelor, all on top of 100 mg aspirin) were included in the index prospective observational cross-sectional study. Written informed consent was obtained from all patients, and the study protocol was approved by the Ethical Review Board of Dong-A University Hospital. Exclusion criteria were DAPT maintenance < 1 year, hemodynamic instability, malignancies, active bleeding or major surgery within 4 weeks, severe chronic renal failure, treatment with other types of antiplatelet agents (e.g. cilostazol, or glycoprotein IIb/IIIa receptor blocker). Clinically relevant bleeding complications were recorded by BARC type ≥ 2 scale Mehran et al., 2011 within 1 month, and then at 1 year of follow-up. The primary endpoint was predictive value of the CRUSADE bleeding score versus residual platelet reactivity measured by VerifyNow Analyzer at 1 month and at 1 year post-stenting. There were no secondary endpoints in the study.

### 2.2 Samples

Blood samples were obtained by venipuncture after a 2 ml discard sample, and were drawn into Greiner Bio-One 1.8 ml Vacuette blood collection tubes containing 3.2% citrate (Greiner Bio-One, Monroe, NC, USA). The whole blood citrate mixture was used for VerifyNow Rapid Platelet Analyzer. Blood was collected between 4 and 12 h after last administration of routine medications including antiplatelet agents in the maintenance DAPT phase to reduce the variability during the loading phase. Every measurement was done in duplicate with the mean calculation, but in case of measurements with more than a 20% difference of the mean curve from at least one curve or the correlation coefficient < 0.98 resulted in the measurement being discharged and testing being performed again.

### 2.3 VerifyNow Platelet Analyzer

The P_2_Y12 platelet reactivity assay (Accumetrics, San Diego, CA, USA) is a whole-blood, cartridge–based, optical detection system designed to measure aggregation (Malinin et al., 2007). The ADP receptor binding is measured in a cartridge channel with the presence of a platelet agonist and prostaglandin E1 (PGE1), a suppressor of intracellular free calcium levels which reduces the nonspecific contribution of ADP binding to P_2_Y1 receptors. The test cartridge contains a lyophilized preparation of human fibrinogen coated beads, platelet agonist, buffer, and preservative. Fibrinogen-coated microparticles are used in the VerifyNow-P_2_Y12 cartridge to bind to available platelet receptors. When the activated platelets are exposed to the fibrinogen-coated microparticles, agglutination occurs in proportion to the number of available platelet receptors. This particular analyzer is designed to measure this agglutination as an increase in light transmittance. The whole blood citrate mixture was added to the cartridge, and agglutination between platelets and coated beads is recorded. VerifyNow-P_2_Y12 assay results are expressed in Platelet Reaction Units (PRU). An electronic quality control, positive and negative control tests were performed on each instrument every day prior to performing any patient samples. The internal electronic quality control device, kits with negative and positive control test were provided by the assay manufacturer.

### 2.4 Statistical Analysis

Continuous variables are expressed as means ± standard deviations and were analyzed using Student's *t*-test. Categorical variables were summarized in terms of numbers and percentages, and were compared by using chi-square test or Fisher exact test. Univariable and multivariable Cox proportional hazard regression were used to determine independent factors associated with incidences of variables. All data with a p value < 0.2 in the univariable analysis were then entered into a multivariable model. The ability of the assay to discriminate between patients with and without bleeding at 1 month and at 1 year was evaluated by ROC curve analysis (using MedCalc Version 12.2.1, MedCalc software, Mariakerke, Belgium). The prognostic utility of the dual methods has been assessed by c-statistic estimates. A p value < 0.05 was considered to indicate significance. Categorical variables are summarized as frequencies with percentages, and continuous variables as mean values with standard deviation. Between-group comparisons were performed by using the Pearson's chi-square test or Fisher's exact test for categorical variables, and by using the one-way analysis of variance or Kruskal–Wallis test for numerical variables, as appropriate. Inter-Assay variability (40.4%) and Intra-Assay variability (24.0%) for platelet reactivity indices were also assessed. The CV value may be large due to the time difference between the two measurements. Statistical analyses were performed using SPSS version 18.0 (SPSS Inc., Chicago, IL, USA).

## 3 Results

The baseline demographics and clinical characteristics of the entire patient pool dependent on experiencing bleeding event are presented in Table 1. Background clinical variables and admission biomarkers were distributed differently depended heavily on future bleeding events. In fact, patients experienced bleeding were older, more frequent females, and treated with newer P2Y12 inhibitors (prasugrel and ticagrelor) than after clopidogrel. Delayed bleeding risks were also associated with diabetes, hypertension, and prior stroke. Among acute coronary syndromes, NSTEMI and STEMI patients, but not unstable angina were linked with more bleeding. Admission biomarkers were not particularly useful for dichotomization, with the exception of diminished glomerular filtration, which was lower in those who bleed.

**Table 1:** Baseline demographics and clinical characteristics dependent on bleeding.

Variables	Non-bleeding (n = 749)	30-days bleeding (n = 113)	1-year bleeding (n = 41)	p-value
Age, year^a^	64.2 ± 10.3	72.5 ± 8.4	69.5 ± 10.4	0.006
Female, n (%)	198 (26.4)	58 (51.3)	14 (34.1)	0.02
BMI, kg/m^2^	24.6 ± 3.1	23.6 ± 3.4	23.9 ± 3.4	NS
Admission diagnosis, n (%)				
Unstable angina	532 (71.0)	54 (47.8)	21 (51.2)	0.002
NSTEMI	193 (25.8)	49 (43.4)	17 (41.5)	0.0001
STEMI	24 (3.2)	10 (8.8)	3 (7.3)	0.0001
P2Y12 inhibitors, n (%)				
Clopidogrel	713 (95.2)	105 (92.9)	39 (95.1)	NS
Prasugrel	32 (4.3)	5 (4.4)	2 (4.9)	NS
Ticagrelor	4 (0.5)	3 (2.7)	0 (0)	0.001
Risk factor, n (%)				
Diabetes mellitus	307 (41.0)	59 (52.2)	21 (51.2)	0.045
Hypertension	438 (58.6)	91 (80.5)	31 (75.6)	0.01
Dyslipidemia	468 (62.5)	62 (54.9)	26 (63.4)	NS
Current smoking	180 (24.1)	18 (15.9)	8 (19.5)	NS
Past history, n (%)				
Prior MI	171 (22.9)	30 (26.5)	11 (26.8)	NS
Prior PCI	308 (41.2)	49 (43.4)	15 (37.5)	NS
Prior stroke	63 (8.4)	22 (19.5)	6 (14.6)	0.001
Heart rate, bpm	73.3 ± 13.8	85.5 ± 19.2	78.9 ± 20.0	NS
Systolic BP, mm Hg	129.7 ± 21.7	132.2 ± 27.0	130.8 ± 28.4	NS
LVEF, %	59.7 ± 10.5	55.5 ± 13.1	55.2 ± 11.4	NS
Total cholesterol, mg/dl	163.4 ± 39.0	167.3 ± 49.8	163.1 ± 47.1	NS
HbA1c, %	6.6 ± 1.3	6.7 ± 1.3	6.8 ± 1.3	NS
Platelets count, 10^3ul^	210.8 ± 58.4	205.8 ± 75.0	233.2 ± 83.6	NS
Hematocrit, %	37.9 ± 4.7	31.4 ± 5.5	36.4 ± 5.1	NS
eGFR, ml min^− 1^, 1.73 m^− 2^	79.2 ± 24.1	58.6 ± 29.6	68.5 ± 30.4	0.01
Discharge medication (%)				
Statins	458 (61.5)	65 (58.0)	28 (70.0)	NS
CCB	340 (45.6)	56 (50.0)	21 (52.5)	NS
ACEi/ARB	233 (31.2)	41 (36.6)	7 (17.5)	0.02
Beta blockers	440 (59.1)	65 (58.0)	18 (45.0)	0.007

a Between bleeding and non-bleeding groups; BMI – body mass index; CABG – coronary artery bypass grafting; CKD – chronic kidney disease; LVEF – left ventricular ejection fraction; Hb-hemoglobin; eGFR – estimated glomerular filtration rate; CCB – calcium-channel blockers; ACEi - angiotensin-converting-enzyme inhibitor; ARB – angiotensin receptor blockers; NS – not significant.

Both residual platelet reactivity and CRUSADE score were higher in patients who experienced bleeding event at 30-days, but not significantly different for 1-year bleeding when compared with no bleeding cohort. Additional statistical considerations, including assessing area under the curve are presented in Table 2.

**Table 2:** Area under the curve for VerifyNow and CRUSADE for major bleedings.

Variables	AUC (95% CI)	Z statistics	p value
30-days follow up			
VerifyNow	0.605 (0.572–0.637)	3.372	0.0007
CRUSADE score	0.816 (0.789–0.840)	15.775	< 0.0001

1-year follow up			
VerifyNow	0.546 (0.512–0.579)	1.566	0.1173
CRUSADE score	0.755 (0.726–0.783)	11.154	< 0.0001

**Fig. 1:**
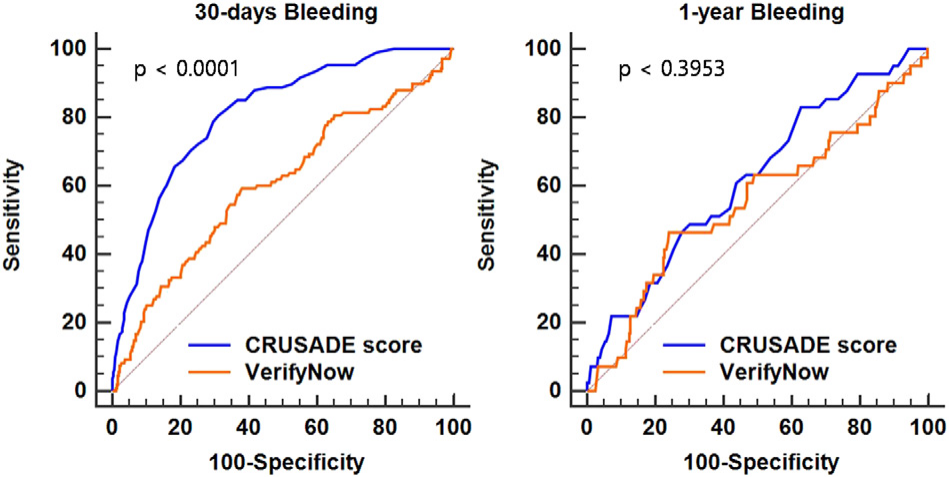
ROC curve analysis for predicting major bleedings.

**Fig. 2:**
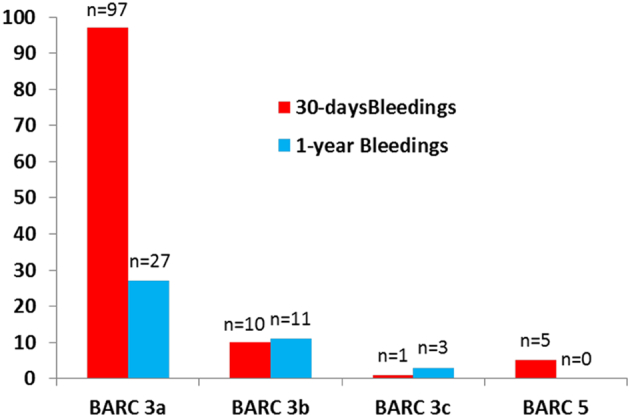
Distribution of major bleeding events by BARC scale.

**Fig. 3:**
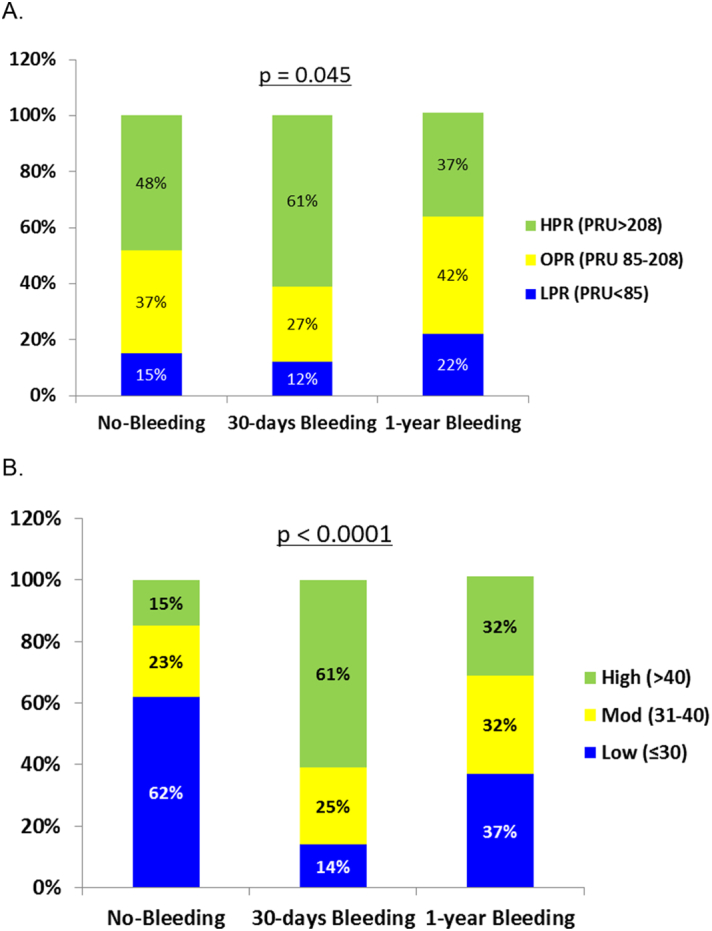
Distribution of PRU values (A) and CRUSADE score (B) for major bleeding rates with regard to risk categories.

Importantly, multivariate adjusted model revealed that CRUSADE score was superior to platelet reactivity values for 30-days bleeding events. The distribution of BARC classification bleeding scores in presented in Fig. 1. The curve analyses (Fig. 2), and spread of platelet reactivity and CRUSADE scores dependent on bleeding are presented in Fig. 3A and B respectfully.

## 4 Discussion

There are four most important findings which can be yielded from the index study. First, major bleeding events on DAPT are much more common in the real-life setting than reported in the published randomized trials, at least in East Asians. Second, the CRUSADE score was superior to assessing residual platelet reactivity while on DAPT for predicting bleeding. Third, CRUSADE may be successfully applied not only for predicting in-hospital bleeding, as originally designed, but expanded for the entire first month post-stenting including many outpatients. Finally, while such predictions at admission for index ACS were quite reliable for 30-days hemorrhages, but both admission platelet testing or CRUSADE were not useful for the delayed 1-year risks. Recently, there was an explosion of publications regarding the monitoring of platelet activity concerning the impact of determined cut-off values on clinical outcomes, especially in patients with acute coronary syndromes treated invasively (e.g. Brar et al., 2011, Parodi et al., 2011, Breet et al., 2010, Guo et al., 2016). The main objective of these studies was to define the incidence of thrombotic occlusions or bleeding by linking such adverse events with residual platelet reactivity. Some evidence emerged lately assessing the risk of bleeding and specific cut-off values for its predictability in patients after acute coronary syndromes (Généreux et al., 2015, Aradi et al., 2015), and those undergoing heart surgery (Mishra et al., 2015, Kuliczkowski et al., 2015). The data on applying CRUSADE success are somewhat mixed for two main reasons. First, patients differ substantially, somewhat neglecting that this useful score was designed exclusively for non STEMI cohort predicting very early in-hospital major bleeding. Second, there are over dozen current bleeding classifications, and their inventors may be biased in promoting their own scales at expense of other useful algorithms. (Biancari et al., 2017). Some other integrative models, such as HASBLED are much more simple than CRUSADE, and unclear how they may be implemented for the similar delayed approach to pick up either bleeding or adverse thrombotic events (Hsieh et al., 2017). Expanding original CRUSADE applicability beyond exclusive non STEMI patients (Subherwal et al., 2009) to the entire post-ACS pool is also important, especially considering similar to our data yielded from Egyptian patients (Al-Daydamony & Farag, 2016). Another interesting study suggests some benefit of combining CRUSADE with platelet activity testing for yielded more accurate predictive value for 1-year bleeding risk, which was not achieved in our study. (Li et al., 2016). Overall, the available evidence suggests that mainstream use of platelet analyzers may be less reliable than clinical models such as CRUSADE to assess individual bleeding risk, and should not be currently recommended (Reed et al., 2015, Lordkipanidzé et al., 2008) what is in full agreement with the index data. Our results are also in agreement with another elegant study suggesting that both conventional aggregometry and VerifyNow tests were not particularly useful to identify patients at higher risk of bleeding (Fattorutto et al., 2003). Finally, (Garay et al., 2016) indicate the special difficulties in delayed bleeding prediction matching well with the index data. That message is particularly critical since late catastrophic hemorrhages are usually the most deadly, unexpected, and hard to prevent.

### 4.1 Strengths and Limitations

Large sample size, applying reasonably validated uniformed bleeding CRUSADE score with simultaneous assessment of platelet activity by reliable established platelet test, with very careful follow-up are obvious assets. Single busy clinical centre environment also reduce variability of techniques and outcomes. Each bleeding case was clinically verified and confirmed. Finally, we applied the novel BARC bleeding classification Li et al., 2016, which was introduced to fairly and objectively count hemorrhages. This scale have consistent practical trouble to be validated in the major DAPT trials, since the industry prefer to hide, rather than to objectively report bleeding rates, therefore, utilizing conservative exclusive TIMI or GUSTO scales to minimize the risks. There are certain limitations worth mentioning. It should be emphasized that there might be important confounders to our analysis potentially impacting the conclusions including potentially missed bleeding events. Among most important limitations are non-randomized observational cross-sectional design, and single platelet activity assessment. In addition, the background differences among the patients, and pooled analyses of various stenting techniques also limit the applicability of the index dataset. We used a “real-life” registry, acknowledging that minority of prasugrel and ticagrelor use may compromise the homogeneity of clopidogrel data, potentially increasing the statistical “noise”. Another shortcoming is the fact that we did not capture minor bleeding events, limiting the clinical applicability of the index dataset. Future studies should definitely focus more on minor haemorrhagic complications, which are critical for compliance, and drug discontinuations. In this study we deliberately scope on delayed bleeding risks, also acknowledging that most bleeds occur early, and those were missed since we used up to 12 months lag in capturing events. From a pragmatic point of view, it will be necessary to determine whether the additional cartridge for assessing aspirin in combination with clopidogrel cartridge may improve the prognostic value of platelet testing. This is especially true since applying DAPT strategy, both antiplatelet agents may exhibit response variability, and impact bleeding risks. Considering recently discovered association of malignancies, potency of antiplatelet agents, and bleeding, we now feel that it was a mistake to exclude cancer patients from this registry.

In conclusion, major bleeding events are more common in real life than reported in clinical trials. The CRUSADE score was superior to platelet testing for predicting short-term, but not 1 year bleeding in Korean patients undergoing percutaneous coronary intervention and treated with DAPT. Further evidence should be urgently retrieved from large unbiased national registries or insurance claims datasets.

## Funding

This research was supported by a grant from the Korea Health Technology R&D Project through the Korea Health Industry Development Institute (KHIDI), funded by the Ministry of Health & Welfare, Republic of Korea (grant number: HI14C1731) and funded by the Ministry of Education, Science and Technology (NNRF-2015R1D1A1A09057025) to MHK. Part of this work was supported by the “Brain Pool” program funded by the Korean Ministry of Science and Technology to VS.

## Author Contributions

Study design: JB, SYC, MHK.

Data collection: JB, SYC, MHK.

Data analysis: MHK, VS.

Literature search: SYC.

Figures: JB.

Data interpretation: MHK, VS.

Manuscript writing, original draft: JB, MHK.

Manuscript writing, review and editing: JB, MHK, VS.

## Conflicts of Interest

None of the authors have any personal or financial conflicts of interest in relation to the data presented in this study.
